# High yield strategies for triterpenoid biosynthesis in cell factories

**DOI:** 10.71150/jm.2509018

**Published:** 2026-04-21

**Authors:** Mingzhu Zheng, Chuang Liu, Ceyuan Liu, Jing Xie, Gen Pan, Can Zhong, Jian Jin

**Affiliations:** 1 Hunan Academy of Chinese Medicine, Hunan University of Chinese Medicine, Changsha 410036, P. R. China; 2 Institute of Chinese Medicine Resources, Hunan Academy of Chinese Medicine, Changsha 410013, P. R. China

**Keywords:** triterpenoids, biosynthesis, yeast, enzymes, strategies

## Abstract

Triterpenoids are natural products widely found in the plant kingdom and have various pharmacological effects such as anti-inflammatory, antioxidant and anti-tumour. However, the content of triterpenoids in medicinal plants is low, and it is difficult to purify and isolate them due to their complex structure. The efficient production of some triterpenoids in chassis organisms has been achieved by constructing a heterologous triterpenoid synthesis pathway in engineered strains such as yeast, modifying the key enzymes in the pathway, and adjusting the metabolism of yeast. Modification of key enzymes in the synthetic pathway is currently an effective strategy to enhance the heterologous synthesis of triterpenoids. This paper reviews the current research progress on the modification of key enzymes downstream in the synthetic pathway and the design of key enzymes around them to enhance triterpenoid production in five main areas: 1) increasing the supply of triterpenoid precursors; 2) inhibition of the natural sterol pathway; 3) fusion expression of related enzymes; 4) compartmentalisation of the metabolic pathway; and 5) tapping and enhancing the triterpenoid efflux pump. Finally, recent advances and applications of artificial intelligence (AI) in enzyme engineering and pathway design for triterpenoid biosynthesis are highlighted. Challenges and perspectives for further increasing the yield of triterpenoid synthesis in *Saccharomyces cerevisiae* are presented.

## Introduction

**Fig. 1.**
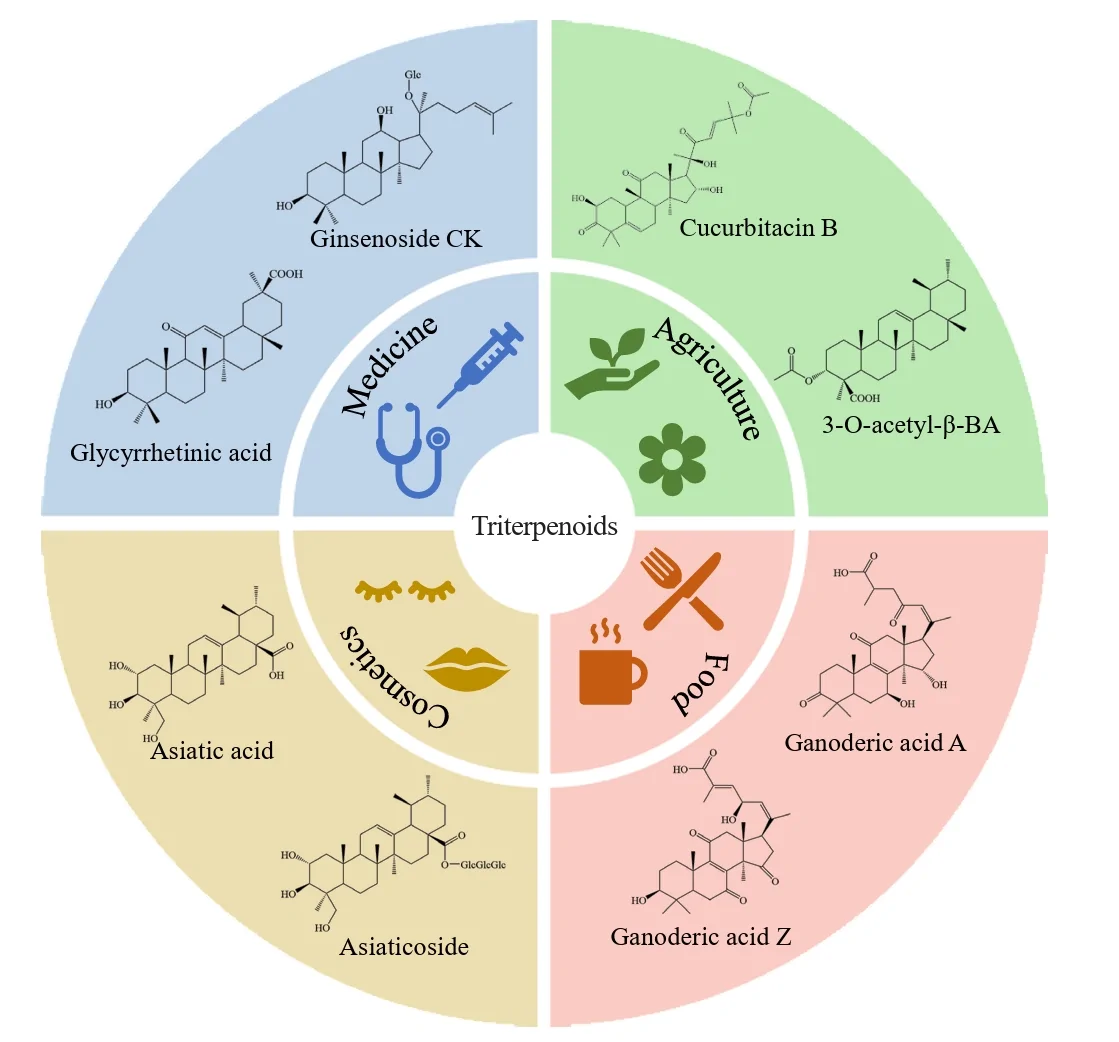
Wide range of applications of triterpenoid compounds.

Triterpenoids, a class of terpenoids consisting of six isoprene units (C30), widely distributed in plants, animals and marine organisms, especially in dicotyledonous plants with the largest distribution, usually in free form or combined with sugar to form a glycoside or ester form (Hamberger and Bak, 2013), most of which are soluble in water, and the aqueous solution produces a large number of persistent soap-like bubbles after shaking, so it is also known as triterpenoid saponins. Six isoprene units make up squalene, which is converted to 2,3-oxo-squalene, which is then cyclised to form different triterpenoid matrices, which can be further oxidised or modified by acylation or glycosylation, and a small proportion of triterpenoids can be generated directly from squalene (Guo et al., 2020). These compounds are widely used as plant secondary metabolites in medicine, food, agriculture, and cosmetics (Fig. 1). In recent years, more and more triterpenoids have attracted the attention of researchers as the main active ingredients of many traditional medicines, e.g., ginsenosides possess a variety of pharmacological activities including anti-inflammatory (Gao et al., 2020), anticancer (Sun et al., 2017), antidiabetic (Zhou et al., 2019), and cardiovascular protection (Fan et al., 2020; Sarhene et al., 2021). Betulinic acid significantly inhibits the growth and glycolytic activity of breast cancer cells and promotes apoptosis of cancer cells, as well as effectively inhibits metastasis of cancer cells, and no adverse effects have been observed in spontaneous models of breast cancer and in zebrafish breast cancer xenograft model (Aly et al., 2024).

Typically, triterpenoids are mainly derived from medicinal plants. However, medicinal plants tend to be slow-growing and take a long time to accumulate active ingredients. In addition, the diversity of triterpenoid structures makes it very difficult to isolate them in the presence of structurally or polarity-wise similar congeners, especially triterpenoids that are naturally low in abundance, and these factors limit the availability of triterpenoids from plant sources. The presence of highly fused polycyclic systems with multiple chiral centres in natural triterpenoids and their artificial derivatives also makes their chemical synthesis pathways laborious and time consuming as well (Jauch, 2008; Tong et al., 2021). In contrast, biosynthesis of active ingredients using microorganisms has the advantages of short synthesis cycle, controllable quality and environmental friendliness, which provides an effective strategy for the sustainable development of active ingredients in medicinal plants.

To date, triterpenoid biosynthesis has been realised in a wide range of microorganisms, such as two classical representatives of chassis organisms: *Escherichia coli* and *Saccharomyces cerevisiae*. *S. cerevisiae* can provide 2,3-squalene oxide for triterpenoid synthesis through its own pathway and has a complex post-translational modification and organelle system that favours the expression of cytochrome monooxygenases (CYP450s) necessary for the biosynthesis of many plant secondary metabolites, and is therefore often used as a chassis organism for the synthesis of complex plant secondary metabolites.

## Biosynthetic Pathways of Triterpenoids

The process of triterpenoid biosynthesis can be divided into two main stages: (i) the synthesis of the common precursor of triterpenoids, 2,3-oxidosqualene (Fig. S1); and (ii) the cyclisation of 2,3-oxidosqualene to form the triterpenoid backbone, which is then further modified by other enzymes (Fig. S2). In general, there are two main synthetic pathways for 2,3-epoxy squalene: the mevalonate pathway (MVA) and the methylerythritol phosphorylation (MEP) pathway. In plant cells, the MVA pathway mainly exists in the cytoplasm and endoplasmic reticulum and is involved in the synthesis of sterols, sesquiterpenes, and triterpenoids; the MEP pathway mainly exists in the plastids of plant cells and is mainly involved in the synthesis of monoterpenes, diterpenes, and isoprenoids, etc. (Luo et al., 2016), but in *S. cerevisiae*, the 2,3-epoxysqualene is mainly derived from the MVA pathway. The MVA pathway yields isopentenyl diphosphate (IPP) and dimethylallyl diphosphate (DMAPP) as its final products. Farnesyl diphosphate synthase (FPS) then condenses one DMAPP molecule with two IPP molecules to form farnesyl diphosphate (FPP). Subsequently, two molecules of FPP undergo a head-to-head condensation reaction, catalyzed by squalene synthase, to form squalene (SQ). Finally, squalene epoxidase (SE) introduces an oxygen atom across the central C=C double bond of squalene, producing the key triterpenoid precursor, 2,3-oxidosqualene. The latter is catalysed by squalene epoxidase (SE), which inserts an oxygen atom between the C=C double bonds to produce 2,3-epoxy squalene. 2,3-Epoxysqualene is cyclised by different epoxysqualene cyclases (OSCs) to generate various triterpenoid skeletons, and then chemical modifications such as oxidative replacement and glycosylation of the triterpenoid skeletons are successively carried out by cytochrome P450 monooxygenases (CYP450s), glycosyltransferases (UGT), and glycosidases, etc., to finally obtain different types of triterpenoid products. In *S. cerevisiae*, lanosterol is the only product of the cyclisation of 2,3-epoxy squalene, which is also an important precursor of the sterol pathway (Guo et al., 2020).

## Strategies for Improving Triterpenoid Biosynthesis

At present, many key genes of triterpenoid synthesis have been identified and heterologously expressed in yeast, but it is often difficult to obtain satisfactory titer only by introducing the triterpenoid synthesis pathway to the chassis organisms. Metabolic engineering can increase the production of target products and reduce the generation of by-products, and also improve the cell viability of chassis organisms. It is a powerful means in heterologous biosynthesis. By means of modular engineering, promoter engineering, protein engineering, transporter engineering, and other means in metabolic engineering, many chassis have been biologically modified to produce various triterpenoids under different conditions. Table 1 summarizes key production metrics for representative triterpenoids and Table 2 lists some specific strategies to improve the efficiency of triterpenoid synthesis.

### Modularization of metabolic pathways

The balance of metabolic flux in microbial cells is usually maintained by a very complex and tight regulatory system. The modification of endogenous genes and heterologous insertion into recombinant strains often lead to metabolic imbalance, resulting in growth retardation and insufficient production of target compounds (Wu et al., 2016). Especially for some compounds that need long and complex synthetic pathways, because the overexpression of a large number of pathway genes will produce excessive metabolic pressure. Modular engineering provides a new strategy for the construction and system optimization of natural product engineering strains. The whole synthesis pathway of the target product is divided into a series of engineering modules, and the expression level of each module is coordinated to achieve the global adjustment of the whole pathway and guide the metabolic flux to the synthesis of the target product. A key application of modular engineering is the fine-tuning of gene expression at the transcriptional level to balance pathway fluxes. This can be achieved by constructing libraries of promoters with varying strengths and systematically testing different promoter-gene combinations upstream of pathway genes. High-throughput screening of the resulting strain library allows for the selection of strains exhibiting balanced metabolic flux and improved product formation (Du et al., 2012). While transcriptional tuning is common, fine-tuning at the translational level (e.g., through ribosome binding site engineering or codon usage optimization) also offers precise control over enzyme abundance and warrants consideration in modular design strategies. At present, modular engineering has been successfully applied to the construction of natural product synthesis engineering strains. For instance, Wang et al. (2019) leveraged modular engineering to optimize the synthesis of ginsenoside Rh2 in *S. cerevisiae*, significantly improving the titer of this valuable compound. Xiu et al. (2022) divided the 7-DHC synthesis pathway into central metabolism module, MVA module, squalene module and 7-DHC module, and reconstructed the de novo synthesis pathway of 7-DHC in *S. cerevisiae*. By weakening the carbon metabolic overflow in the central metabolism module, removing the key speed limiting steps in the MVA and squalene modules, increasing the precursor supply of 7-DHC module and other strategies to optimize the module, and combining with the dynamic control tools to carry out the collaborative balance of the overall synthesis pathway of 7-DHC, an efficient production engineering strain with 7-DHC yield of 2,870 mg/L was obtained.

### Increase the supply of precursor substances

Increasing the supply of precursor substances is currently a commonly used method in metabolic engineering. Squalene is a key precursor in the upstream synthesis of triterpenoids, and the reduction of hydroxymethylglutaryl-coenzyme A (HMG CoA) to methylglutarate catalyzed by hydroxymethylglutaryl-coenzyme A reductase is generally considered the rate limiting step in the MVA pathway. Overexpression of the truncated HMG1 gene encoding HMG CoA (tHMG1) can lead to the accumulation of squalene, which is highly beneficial for the synthesis of triterpenoids. Overexpression of ERG1, which catalyzes the oxidation of squalene to 2,3-epoxysqualene, increased the production of protopanaxadiol by 10 times (Dai et al., 2013), indicating that ERG1 may be another rate limiting enzyme in the triterpenoid synthesis pathway.

A critical precursor for the MVA pathway is acetyl-CoA. Rewiring central carbon metabolism to enhance acetyl-CoA supply represents a powerful strategy. For example, Meadows et al. (2016) engineered *S. cerevisiae* to reduce flux toward acetate, a competing pathway, thereby redirecting carbon toward acetyl-CoA and significantly boosting the production of isoprenoid precursors. Such engineering of precursor supply and redox balance is crucial for high-level terpenoid biosynthesis.

In addition, increasing the expression of all enzymes in the MVA pathway is also beneficial for increasing carbon flux in the MVA pathway. In brewing yeast, multi copy expression of 10 genes involved in the mevalonic acid pathway resulted in the production of up to 18 g/L of damaxanediol type triterpenoids (damaxanediol II and protopanediol) (Wang et al., 2019). However, similar strategies do not always have a significant impact, such as the reported production of Damatane type triterpenoids being much higher than that of oleanolic acid and ursolic acid. The difference in yield may be caused by the varying activities of different OSCs. However, the high-level expression of rate limiting enzymes and other enzymes in the synthesis pathway is considered a necessary and effective strategy for increasing triterpenoid production.

### Inhibition of the natural sterol pathway

*S. cerevisiae* has become the most commonly used chassis organism for most heterologous synthesis due to the advantages of its abundant metabolic pathways and the structural and functional integrity of its organelles. However, competition between its intracellular native and heterologous synthetic pathways has been considered as an obstacle to achieve high yields of target products. Strategies to down-regulate protein activity in the native pathway of *S. cerevisiae* have been widely used to increase the yield of various target products. The sterol pathway has been the main competing pathway for triterpenoid synthesis, and down-regulation of the activities of some enzymes in the sterol pathway has been widely used to increase terpene yields. Paddon et al. (2013) inhibited the expression of ERG9 by replacing its natural promoter with a copper-regulated promoter, effectively targeting carbon flow to artemisinic acid production. Peng et al. (2018) investigated an N-deferred iron-dependent protein degradation strategy and sterol-responsive transcriptional regulation to down-regulate Erg20p activity, which could significantly increase the production of monoterpenes (limonene or geraniol) in engineered *S. cerevisiae*.

Similarly, a number of protein down-regulation strategies have been applied to Erg7p. Bröker et al. (2018) used a copper-regulated promoter to repress the expression of the *ERG7* gene, which was significantly repressed in the presence of 150 μM CuSO_4_, at which point 2,3-oxidised squalene’s significantly accumulated. However, the accumulated 2,3-oxidised squalene’s appeared to contribute little to the increased triterpenoid yield. Zhao et al. (2019) used the *TetR-TetO* gene regulatory system to fine-tune the expression of the lanosterol synthase gene (*ERG7*), which further led to an increase in PPD yield by about 10%.

### Fusion expression of related enzymes

Previous studies have shown that end-to-end multi protein fusion can make different enzymes close to each other and improve their performance. This process allows intermediate products to be directly transferred from one enzyme to the next, bypassing the need for long-distance diffusion and transportation, and minimizing the accumulation of intermediate products and unwanted side reactions. It is a direct and effective yield increase strategy (Guo et al., 2024b). For example, the direct fusion expression of the two enzymes through protein linker (GGGGS*3) can effectively improve the substrate conversion rate, and this strategy has been successfully applied to the optimization of the synthesis pathway of flavonoids (Liu et al., 2021) and other substances in *S. cerevisiae*. Liu et al. (2019) fused expression of *erg20* and farneside synthase in *Yersinia lipolytica* can increase the titer of α-farneside by 4.8 times, and the titer of strain F2 can reach 0.6 g/L. In the production of terpenes, Xu et al. (2022) promoted the interaction between *CYP* and *CPR* by fusion expression of *CYP* and *trcpr1* (truncated CPR) in steviol synthesis, and obtained the group with the highest steviol yield in this module.

The success of enzyme fusion is highly dependent on the design of the fusion architecture. Critical parameters include the relative orientation of the enzymes and the length and composition of the peptide linker connecting them. Short linkers often enhance pathway efficiency by promoting close proximity, while the amino acid composition at the linker termini, particularly the first position where alanine is frequently favorable, can strongly impact fusion performance (Bouin et al., 2023). These design principles, initially explored in terpenoid pathways in *E. coli*, provide valuable guidance for constructing functional fusions in yeast.

Linker plays an important role in enzyme fusion. Linker can be divided into three categories according to its structure: flexible linker, rigid linker and in vivo cleavable linker. The most common flexible junction is (GGGGS)_n_, which is composed of Gly and ser residues; Sequence (EAAAK)_n_ forms a rigid link in the form of α-helix, which can effectively maintain the distance between enzymes. Length and flexibility are the first factors to be considered when selecting linkers. Adjusting the copy number of linkers "n" can change the length of linkers to optimize the distance between fusion proteins, and this length will also affect the distance between domains in the three-dimensional structure (Huang et al., 2021).

**Fig. 2.**
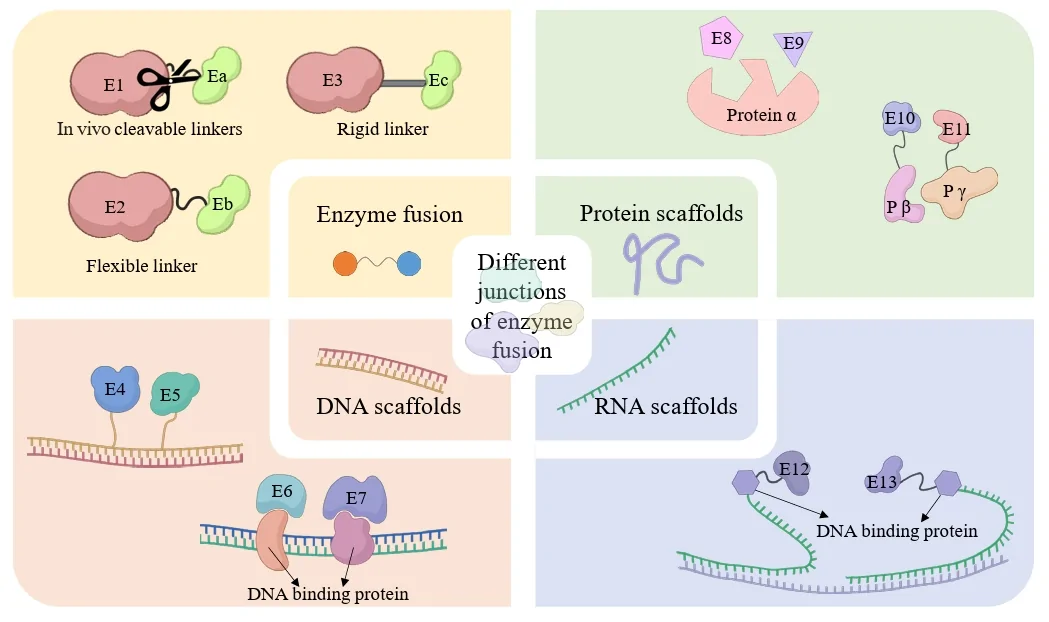
Different ways of enzyme fusion.

In addition to linker, protein, DNA or RNA can also be used as scaffolds to form a more stable spatial arrangement between enzymes. A key feature of synthetic protein scaffolds is to control the stoichiometry of spatial tissues and enzymes, and to rescue intermediates from diffusion or competition. RNA scaffolds are easier to design and express in vivo than protein scaffolds, and cause less metabolic burden (Pothoulakis et al., 2022). The structure of DNA scaffold has predictable local geometry, and its stability in vivo is usually sequence independent, allowing the creation of different scaffold structures without compromising its availability. In addition, a large number of DNA binding proteins exist in nature, which enhances the versatility of DNA scaffolds (Guo et al., 2024b). Figure 2 shows the different connections between related enzymes. Selecting the appropriate fusion method can bring a variety of advantages to the expression of fusion protein, such as improving structural stability, enhancing biological activity, improving expression level, changing pharmacokinetics, and achieving in vivo targeting, but sometimes it will also affect the localization or catalytic activity of the enzymes involved in the fusion, which must be considered before fusion.

### Compartmentalization of metabolic pathways

**Fig. 3.**
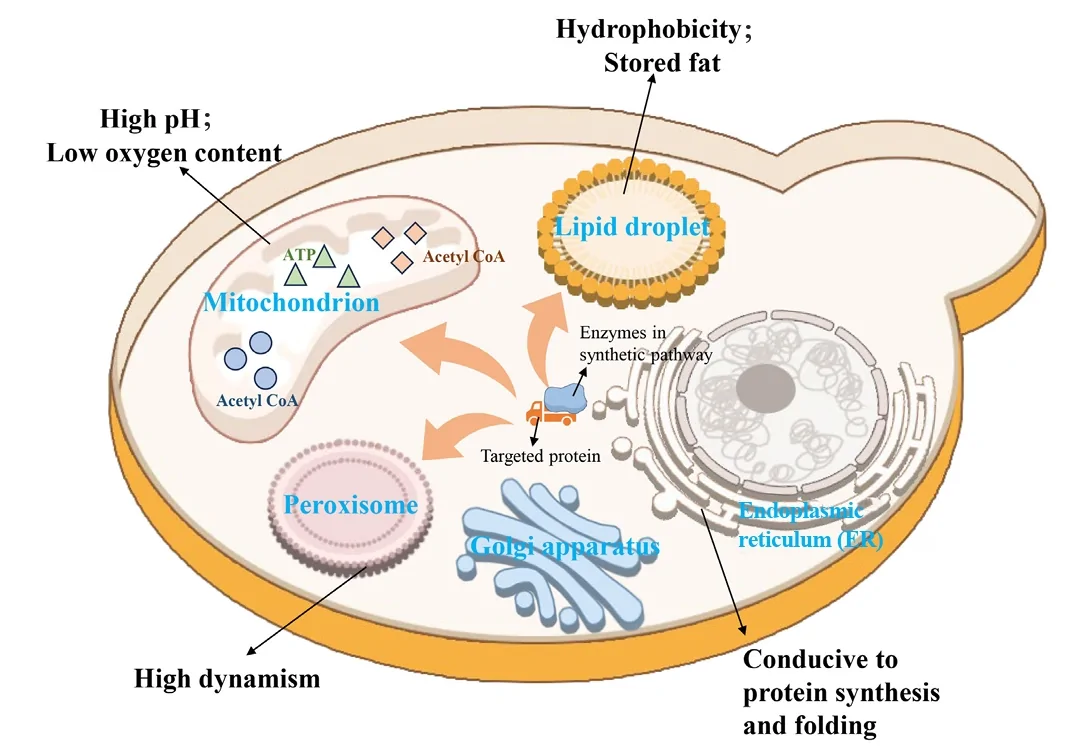
Compartmentalization of metabolic pathways and advantages of biosynthesis of triterpenoids by organelles due to different internal environments.

The metabolic pathways in eukaryotes are usually distributed in different cell compartments, and the concentration and availability of substrates, cofactors, etc. in each organelle are different. In addition, the problem of substance transport between different organelles poses challenges to the regulation of metabolic pathways for synthesizing target products. In response to these issues, strategies have been developed to immobilize enzymes and metabolites in specific organelles to obtain higher concentrations of enzymes, substrates, and cofactors in a small range. In addition, specific environments within each organelle also provide assistance for the synthesis of target products (Guo et al., 2024b). Mitochondria contain a large number of key precursor substances, such as acetyl CoA, ATP, NADH, etc., and have a high pH value and low oxygen content, which is conducive to maintaining a more reducing redox microenvironment, matching the microenvironment of many enzymes with the highest activity, especially those that rely on iron sulfur clusters (ISC) (Hu et al., 2008; Mühlenhoff and Lill, 2000). Lipid droplets are the storage sites of lipids in cells. Figure 3 shows the advantages of triterpenoid biosynthesis by different organelles in yeast. Studies have shown that the enhancement of lipid production capacity in cells will significantly affect the biosynthesis of hydrophobic compounds. Larroude et al. (2018) found that the lipophilicity of carotenoids makes it possible to store them in lipid droplets. When heterologous production of β-carotene in *Yersinia lipolytica*, the lipid producing strain can produce more β-carotene than the wild type. The endoplasmic reticulum is the site of protein synthesis and folding in cells and the best microenvironment for many enzymes related to protein synthesis. By fusing with ER targeted signal peptide, Xiu et al. (2022) localized six enzymes (*erg12*, *erg8*, *mvd1*, *idi1*, *erg20*, and *erg7*) in Er, effectively increasing the titer of 7-DHC to 455.6 mg/L.

### Exploration and enhancement of external discharge pumps

**Fig. 4.**
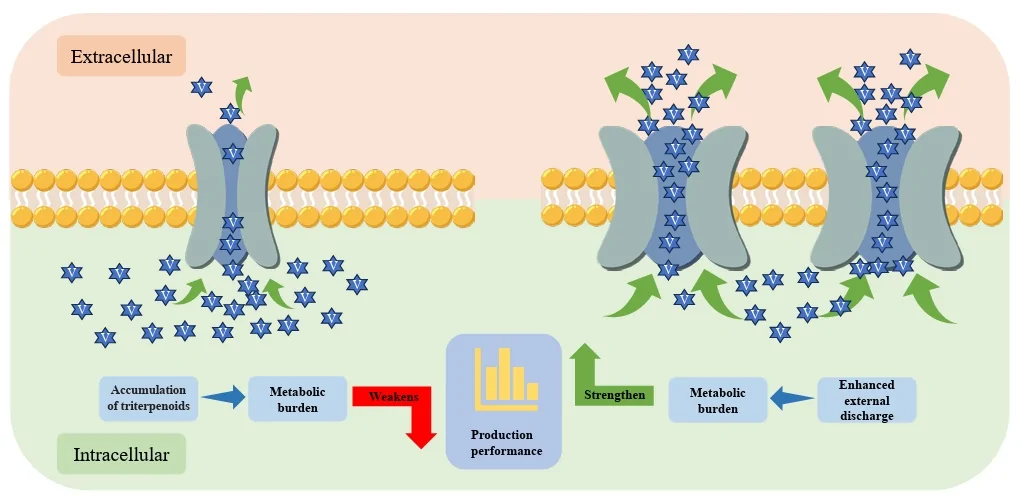
Enhancing the efflux of triterpenoids helps to reduce the metabolic burden of chassis organisms.

Heterologous expression products are often not required for the growth of chassis organisms, and their accumulation in the cell increases the metabolic burden of chassis organisms and weakens their productive performance (Bu et al., 2020; Pereira et al., 2020). The solution means such as adaptive evolution is time-consuming and prone to transmissible contamination in the middle, which is not applicable to all strains. Previous studies have shown that microorganisms can use efflux pumps on the cell membrane to expel drugs, heavy metals, and other substances that are detrimental to cell growth in response to changes in the surrounding environment, which is one of the major reasons for the resistance of microorganisms to antibiotics (da Silva et al., 2023; Engle and Kumar, 2024; Zhang et al., 2024). However, for researchers in synthetic biology, this may provide a good idea to improve the tolerance of chassis organisms. As shown in Fig. 4, with the help of chassis organisms’ own exocytosis mechanism, tapping and strengthening their exocytosis pumps for target triterpenoids so that the triterpenoids can be rapidly excreted out of the cell after synthesis is not only conducive to the isolation and purification of the products, but also reduces the metabolic burden of the chassis organisms and improves their production performance. Efflux pumps, particularly those belonging to the ATP-binding cassette (ABC) and major facilitator superfamily (MFS), can be engineered using several strategies to enhance the export of triterpenoids:

**Heterologous transporter expression:** Introducing efflux pumps from other organisms with known specificity for hydrophobic compounds can improve export. For example, the Arabidopsis ABC transporter AtABCG34 has been demonstrated to export antimicrobial triterpenoids (Kretzschmar et al., 2011). Screening transporter libraries from plants or other fungi for triterpenoid specificity represents a promising approach.

**Promoter tuning and regulatory engineering:** Replacing native promoters with inducible or strong constitutive promoters (e.g., *GAL1*, *TEF1*) can upregulate transporter expression. Huang et al. (2024) used a *GAL* promoter to overexpress 11 ABC transporters in *S. cerevisiae*, identifying PDR11 as the most effective for lycopene efflux, increasing yield by 12.7-fold. However, constitutive high-level expression can impose a significant energetic burden due to ATP consumption, potentially inhibiting growth. The use of dynamic promoters that respond to triterpenoid accumulation or cellular stress may help optimize the trade-off between export efficiency and cellular fitness.

**Membrane lipid engineering:** The activity of membrane-embedded transporters is influenced by lipid composition. Enriching membranes with specific lipids (e.g., ergosterol, phospholipids) to enhance membrane fluidity and transporter integration can improve efflux efficiency. For instance, overexpression of *ERG1* (squalene epoxidase) or *ERG11* (lanosterol demethylase) can modify sterol profiles and potentially improve ABC transporter function.

**Substrate specificity engineering:** Transporters often possess broad substrate ranges. Protein engineering techniques such as directed evolution or rational design based on structural models can be employed to narrow or alter their specificity toward target triterpenoids, thereby reducing the co-export of essential metabolites.

**Balancing export and growth:** While overexpression of efflux pumps can improve product titers, it often reduces cell growth due to ATP drain and potential membrane disruption. Mitigation strategies include using weaker promoters, implementing two-stage cultivation (separating growth and production/export phases), and coupling transporter expression with compensatory pathways for ATP generation.

Combining transcriptomic analysis with experimental validation, Bu et al. (2020) identified SNQ2, an ABC family efflux pump, as the primary mediator of β-carotene secretion in *S. cerevisiae*. Overexpression of SNQ2 increased β-carotene production approximately fourfold. The study also noted that excessive SNQ2 expression could inhibit cell growth, highlighting the need for balanced expression. Similarly, Huang et al. (2024) used an inducible *GAL* promoter to overexpress 11 ABC transporters, finding that the strain overexpressing PDR11 showed the best lycopene efflux performance—12.7-fold higher than the control—resulting in a final total lycopene yield of 343.7 mg/L. These examples provide experimental evidence that transporter overexpression can significantly enhance product yields, while also underscoring the importance of optimizing expression levels to minimize adverse effects on host metabolism and growth.

### Artificial intelligence in triterpenoid enzyme and pathway design

The rapid evolution of artificial intelligence (AI) is redefining the technological frontiers of biosynthesis. Moving beyond general applications, AI is now being deployed to address specific bottlenecks in triterpenoid production, primarily through advanced enzyme engineering and pathway design.

#### 1. AI-assisted enzyme engineering for triterpenoid biosynthesis

Key enzymes in the triterpenoid pathway—such as oxidosqualene cyclases (OSCs), cytochrome P450s (CYP450s), and glycosyltransferases (UGTs)—are prime targets for AI-driven design due to their structural complexity and critical functional roles.

**OSC engineering:** OSCs catalyze the pivotal cyclization of 2,3-oxidosqualene into diverse triterpenoid scaffolds. Traditional mining and mutagenesis approaches are often limiting. Recent large-scale genomic mining, powered by bioinformatics and machine learning (ML), has unlocked tremendous OSC diversity. For instance, Stephenson et al. (2025) mined over 1,000 plant genomes to identify novel OSC families with unique product profiles, providing a rich sequence-function dataset for training predictive AI models. Furthermore, Hakim et al. (2025) employed structure-guided analysis and sequence-based clustering to expand the known stereochemical space of triterpenoids by discovering noncanonical OSCs from green plants. AI can leverage these expansive datasets to predict mutations that alter product specificity or enhance catalytic efficiency. Machine learning models trained on OSC sequence-activity relationships can intelligently guide site-saturation mutagenesis campaigns. Tools like ProteinGAN, a generative adversarial network, can create novel, functional OSC variants by learning from natural sequence landscapes (Repecka et al., 2021). Meanwhile, deep learning models such as AlphaFold2 provide accurate 3D structural predictions for OSCs, enabling virtual screening of mutations that affect active site architecture or substrate channeling prior to experimental validation (Jumper et al., 2021).

**CYP450s and UGT engineering:** CYP450s introduce oxygen functionalities, and UGTs add sugar moieties, both defining triterpenoid bioactivity. Their engineering in microbial hosts is frequently challenged by poor expression, low activity, and undesired regioselectivity. AI-driven rational design offers promising solutions. For predicting substrate specificity, ML models that use protein sequences and reaction features as inputs can identify enzymes likely to catalyze a desired modification (Mellor et al., 2016). DeepEC, a deep learning tool, accurately predicts enzyme commission (EC) numbers from sequence, aiding in the identification of promiscuous enzymes that might accept non-native triterpenoid substrates (Ryu et al., 2019). For enhancing stability and activity, AI-guided directed evolution uses machine learning algorithms (e.g., Gaussian processes, random forest) trained on small mutant libraries to predict beneficial mutations, drastically reducing screening rounds (Yang et al., 2019). This approach has been successfully used to improve properties such as the thermostability of P450s (Romero et al., 2013), a strategy that is directly transferable to triterpenoid-modifying enzymes.

#### 2. AI in metabolic pathway design and optimization

Designing efficient biosynthetic pathways for complex triterpenoids involves navigating vast chemical and enzymatic spaces.

**Retrobiosynthesis and pathway prediction:** AI-powered retrobiosynthesis tools can design novel pathways to target triterpenoids. Template-based approaches like RetroPath RL use reaction rules for enzymatic transformations combined with reinforcement learning to explore plausible biosynthetic routes, considering both reaction feasibility and enzyme availability (Koch et al., 2020). Template-free approaches, inspired by machine translation, utilize models like Transformers to predict precursor molecules directly from the target structure’s SMILES string, enabling the discovery of unprecedented biosynthetic steps (Liu et al., 2017). These tools can propose pathways for triterpenoids not found in nature or suggest optimal heterologous routes.

**Pathway optimization and flux balancing:** Once a pathway is installed, AI can optimize its performance. Generative models like variational autoencoders (VAEs) and generative adversarial networks (GANs) can explore the combinatorial space of promoter strengths, gene copy numbers, and enzyme variants to predict genetic configurations that maximize flux toward the target triterpenoid while minimizing metabolic burden (Gómez-Bombarelli et al., 2018). Reinforcement learning (RL) algorithms can dynamically simulate and optimize metabolic networks, learning policies to adjust enzyme expression levels in silico for maximum yield (Segler et al., 2018).

The convergence of AI with automated high-throughput experimentation is establishing a closed-loop “design-build-test-learn” cycle. AI models propose enzyme mutants or pathway designs, which are rapidly built and tested using automated strain construction and fermentation platforms. The resulting data then feed back to retrain and improve the AI models. For triterpenoid biosynthesis, this paradigm enables the accelerated engineering of high-performance OSCs, P450s, and UGTs, as well as the intelligent design of yeast strains wherein triterpenoid synthesis is optimally balanced with efflux, precursor supply, and cell growth. As natural product databases expand and algorithms grow more sophisticated, AI is poised to transition triterpenoid production from an iterative trial-and-error process to a predictive, rational engineering discipline.

## Challenge and Prospect of Triterpenoid Production by Yeast

In the past decade, researchers have tried various strategies to improve the potency and yield of triterpenoids. Although the yield of each study can not be directly compared with each other due to different chassis organisms and growth conditions, the findings in the research process and the adjustment and combination of various strategies also provide a variety of references for improving the yield of different triterpenoids. At present, the poor production performance and the instability of chassis biosynthesis still make the commercial production of triterpenoid biosynthesis still challenging. A key future challenge lies in the scale-up of triterpenoid production from laboratory to industrial bioreactors. Cells engineered and characterized under well-controlled, homogeneous small-scale conditions may not exhibit the same performance in large-scale fermentations. At larger scales, yeast cells are exposed to spatial and temporal heterogeneities, such as gradients in nutrient concentration, dissolved oxygen, and pH, which can lead to feast-famine cycles and suboptimal metabolic states, ultimately reducing product yields and pathway stability (Haringa et al., 2018). Furthermore, high metabolic stress, potentially from strong induction of heterologous pathways, can lead to the emergence of non-producing but fast-growing subpopulations, a phenomenon reported in terpenoid-producing *E. coli* (Mainali et al., 2026). A similar caveat may apply to yeast systems, given the complexity of triterpenoid biosynthesis. Addressing these bioprocess challenges requires integrating metabolic engineering with bioreactor design and advanced process control strategies.

The competition between triterpenoid biosynthetic pathway and natural metabolic process in yeast is a major obstacle to high-level triterpenoid production. Acetyl coenzyme A plays an important role in all aspects of yeast life activities, which makes the synthesis of triterpenoids compete with other pathways for acetyl coenzyme A all the time, such as histone acetylation, TCA cycle and sterol synthesis (Fig. S3) (Nielsen, 2014). At the same time, the downstream of triterpenoid synthesis also competes with ergosterol biosynthesis for the common precursor 2,3-oxidized squalene. How to use metabolic engineering design to make the metabolic flux flow more to triterpenoid synthesis rather than other pathways without affecting the growth of chassis organisms is particularly important, especially triterpenoid is not necessary for normal yeast growth. Similar strategies have been widely used to modify *E. coli* and *S. cerevisiae* to increase the production of various chemicals, such as 1-butanol (Shen et al., 2011), 1,4-butanediol (Yim et al., 2011), and lactic acid (Lee et al., 2015). In addition, the down-regulation of *erg7* expression can make the metabolic flux more tend to triterpenoid synthesis than ergosterol pathway. However, inhibition of *erg7* alone seems to have a very limited impact on the increase of triterpenoid production. This may be because ergosterol is an important part of fungal cell membrane, which is essential for yeast cell material transport, energy conversion and signal transduction, and *erg7p* has complex interactions with other enzymes involved in sterol biosynthesis (such as *erg6p*, *Erg11p*, and *erg27p*) (Mo and Bard, 2005). For example, the loss of 3-ketosterol reductase (*erg27p*) may lead to the loss of *erg7p* activity at the same time (Mo et al., 2003). Therefore, the strategy of increasing the yield of triterpenoids by inhibiting *erg7p* alone remains to be developed.

Finally, the rapid evolution of artificial intelligence is redefining the technological frontiers of biosynthesis. Through deep convergence with synthetic biology, metabolic engineering, and automation technologies, AI is paving the way for a transformative phase of “intelligent biomanufacturing.” In metabolic pathway optimization, AI architectures leveraging generative adversarial networks (GANs) and variational autoencoders (VAEs) enable the design of synthetic pathways with enhanced yield and reduced metabolic burden by deciphering biosynthetic principles of natural products. Representative examples include the Chemical VAE model developed by Gómez-Bombarelli et al. (2018), which demonstrates unprecedented capability in generating novel metabolic frameworks, and reinforcement learning (RL) approaches (Segler et al., 2018) that dynamically modulate rate-limiting enzyme expression to optimize metabolic flux balance. Furthermore, conducting techno-economic analysis (TEA) will be crucial to assess the economic feasibility of scaling up triterpenoid production. TEA can help identify major cost drivers (e.g., feedstock, separation) and guide research priorities toward achieving commercially viable processes (Sun et al., 2019).

For enzyme functional engineering, the integration of AlphaFold2’s structural predictions, protein language models (e.g., ESM-2), and molecular dynamics simulations provides a robust framework for rationally engineering enzyme variants with non-natural catalytic properties (Stokes et al., 2020). The synergy between AI-driven automated experimentation platforms (Gorgulla et al., 2020) and microfluidics has established closed-loop "design-synthesis-testing-learning" systems. Notably, Synthia’s AI-planned synthetic routes (Mikulak-Klucznik et al., 2020), when coupled with CRISPR-based automated editing tools, can compress the development timeline for complex natural products (e.g., paclitaxel analogs) from decades to mere months. In genome mining, deep learning algorithms like DeepBGC (Hannigan et al., 2019) facilitate efficient identification of cryptic biosynthetic gene clusters (BGCs), while integration with metabolomics enables de novo structural prediction of bioactive compounds (Hoffmann et al., 2022). In the future, with the introduction of causal inference models and quantum chemical calculations (Meijer et al., 2025), AI may break through the complexity limitations of biological systems and achieve a leap from “cell factories” to “programmable biological systems” (Chauhan et al., 2021; Struble et al., 2020).

## Figures and Tables

**Table 1.** Representative examples of triterpenoid production in engineered microbial cell factories

Triterpenoid	Chassis organism	Key strategy	Production method	Titer	Reference
7-Dehydrocholesterol	*S. cerevisiae*	Modular engineering, dynamic control	5 L fed-batch fermentation	2.87 g/L	Xiu et al. (2022)
Protopanaxadiol (PPD)	*S. cerevisiae*	Modular pathway engineering, metabolic flux optimization, Multi Strategy UGT engineering, computational simulation assistance	10 L fed-batch fermentation	11.02 g/L	Wang et al. (2019)
Ginsenoside Rh2	*S. cerevisiae*	Modular pathway engineering, metabolic flux optimization, Multi Strategy UGT engineering, computational simulation assistance	10 L fed-batch fermentation	2.25 g/L	Wang et al. (2019)
Betulinic acid	*S. cerevisiae*	Metabolic engineering, compartment engineering, fermentation optimization	5 L fed-batch fermentation	205.74 mg/L	Tang et al. (2024)
α-Farnesene	*Yarrowia lipolytica*	Optimization of precursor supply, metabolic flow guidance, multi copy integration and fermentation process regulation	1 L fed-batch fermentation	25.55 g/L	Liu et al. (2019)
Squalene	*S. cerevisiae*	The whole squalene synthesis pathway (starting from acetyl coenzyme A) is reconstructed in peroxisome	3 L fed-batch fermentation	32.8 g/L	Ma et al. (2024)
Lycopene	*S. cerevisiae*	Multi module metabolic engineering, dynamic regulation of competitive pathways, cofactor optimization and efflux engineering	50 ml shake flask fermentation	343.7 mg/L	Huang et al. (2024)
Artemisinic acid	*S. cerevisiae*	Engineering transformation and optimization of yeast strains and innovation of fermentation process	Ethanol pulse feeding + IPM extraction fermentation	25 g/L	Paddon et al. (2013)

**Table 2.** Some strategies and specific operations to promote triterpenoid biosynthesis

No.	Strategy	Specific operation	Effect	Reference
1	Increase the supply of precursor substances	Replacement of the native ERG1 promoter with a strong promoter and substitution of endogenous ERG7 with a heterologous ERG7.	Increase the intracellular 2,3-oxidosqualene reserve.	Tang et al. (2024)
2		Utilization of a NADH-dependent HMG-CoA reductase (NADH-HMGR) from *Silicobacterium* malate to replace tHMG1.	Strengthened the MVA pathway activity, improving precursor supply for terpenoid biosynthesis.	Sun et al. (2024)
3	Inhibition of the natural sterol pathway	Site-directed mutagenesis (F699T, I705K) introduced into ERG7.	Shift metabolic flux more towards downstream triterpenoid synthesis rather than sterol pathway.	Guo et al. (2022)
4	Regulating metabolic flux	Knockout of the transcription factor SIP4 regulating gluconeogenesis and ICL1.	Redirected carbon flux from gluconeogenesis toward target product biosynthesis.	Sun et al. (2024)
5		Overexpression of enzymes in the pre-2,3-oxidosqualene pathway.	Elevated cellular sterol content, alleviating growth inhibition caused by sterol depletion and stabilizing membrane integrity.	Guo et al. (2022)
6	Improve the catalytic activity of enzyme	Site-directed mutagenesis (F222Y) introduced into the catalytic domain of CYP450-Uni25647.	Enhanced substrate binding affinity and catalytic activity of CYP450-Uni25647, improving triterpenoid modification efficiency.	Sun et al. (2024)
7		Site-saturation mutagenesis targeting the non-catalytic MXXXXR motif of pentacyclic triterpenoid synthases (PTSs).	Improved catalytic turnover rate by optimizing enzyme conformational dynamics, increasing triterpenoid yield by 18%.	Guo et al. (2024a)
8	Co-expression and fusion expression of related enzymes	Co-expression and fusion expression of CYP450 (BPLO) with cytochrome P450 reductase (CPR, ATR1).	Optimized redox coupling efficiency, reducing electron leakage and increasing hydroxylation activity by 37%.	Tang et al. (2024)
9	Compartmentalization of metabolic pathways	Establishment of an orthogonal acetyl-CoA synthesis shortcut from CO_2_-derived acetate in peroxisomes.	Significantly enhance the accumulation of peroxisome squalene.	Ma et al. (2024)
10		Reconstruction of a complete squalene biosynthetic pathway from acetyl-CoA within peroxisomes.	Relieved the bottleneck of precursor supply and increased the titer of squalene by 4.5 times.	Ma et al. (2024)
11		Co-localization of all enzymes in the squalene-to-betulinic acid pathway to lipid droplets.	The catalytic efficiency was improved by 62% through substrate channel effect.	Tang et al. (2024)

## Supplementary Information

Fig. S1. Generation of 2,3-epoxy squalene during triterpene synthesis.

Fig. S2. Substrate 2,3-epoxy squalene catalysed by different OSCs, CYP/CPR, UGT to generate various triterpenes.

Fig. S3. crossover of acetyl CoA in triterpene biosynthesis and other metabolic pathways in *Saccharomyces cerevisiae*.
